# Kaposi sarcoma in an HIV-infected patient with high CD4 count: a case report and literature review

**DOI:** 10.3389/fmed.2025.1496863

**Published:** 2025-04-25

**Authors:** Kedir Negesso Tukeni, Merid Lemma Kebede, Elias Ababulgu Abadiko, Tamirat Godebo Woyimo, Abdo Kedir Abafogi, Amare Hailu Ashine, Esayas Kebede Gudina

**Affiliations:** ^1^Department of Internal Medicine, Jimma University, Jimma, Ethiopia; ^2^Department of Clinical Radiology, Jimma University, Jimma, Ethiopia; ^3^Department of Pathology, Jimma University, Jimma, Ethiopia

**Keywords:** Kaposi sarcoma, HIV, CD4 lymphocyte count, low-level viremia, human herpesvirus 8 (HHV-8), antiretroviral therapy, Ethiopia, opportunistic infections

## Abstract

Kaposi sarcoma is a tumor commonly associated with human herpesvirus 8 (HHV-8) infection and is more prevalent in individuals with immunosuppression, such as those with HIV/AIDS, organ transplant recipients, or other conditions causing immune dysfunction. It typically presents as purple patches or nodules commonly affecting the skin or mucous membrane of the gastrointestinal tract, and can spread to the lymph nodes and lungs. This case report describes an 18-year-old male with vertical HIV infection, managed on a regimen of dolutegravir/lamivudine/tenofovir (DTG/3TC/TDF), and with a recent CD4 count of 627cells/mm^3^and recent viral load of 378 copies/ml, presented with exacerbation of shortness of breath, productive cough with blood-tinged sputum, and a diffuse skin rash. The patient also had tongue swelling with purple discoloration, as well as swelling of the entire left leg and right arm. On physical examination, the patient appeared acutely ill with marked respiratory distress. Notable findings included a bluish, exophytic mass with irregular borders involving the posterior tongue and the hard palate. There was also non-tender, soft, multiple submental and supraclavicular lymphadenopathy, bilateral crackles on lung auscultation, and widespread, patchy, nodular skin lesions. A high-resolution computed tomography (CT) scan of the lungs was suggestive of Kaposi sarcoma that was confirmed with skin biopsy. The patient was treated initially with antibiotics, and supplemental oxygen, while continuing anti-retroviral therapy. The case underlines that though epidemic Kaposi sarcoma is often a disease of immunosuppressed people caused by retroviral infection, there is still the possibility in those with high CD4 counts despite viral load remains low. A high level of suspicion in high-risk patients presenting with characteristic skin lesions is critical for early diagnosis and prompt initiation of available treatment options for a better patient outcome.

## Introduction

Kaposi sarcoma (KS) is primarily associated with infection by human herpesvirus 8 (HHV-8), also known as Kaposi sarcoma-associated herpesvirus (KSHV). The virus and tumor are substantially more prevalent in certain parts of the world. KS typically affects four distinct populations: those with HIV/AIDS, individuals with organ transplant, the elderly, and those from regions with high KSHV prevalence. The relationship between KSHV infection and the development of KS is not fully understood, although immunodeficiency is known to play a key role. KS typically manifests in individuals with weakened immune systems, such as in those with HIV infection, organ transplant recipient, or the elderly.

The context in which Kaposi sarcoma arises determines the approach to treatment. Epidemic, HIV infection-related KS is widespread in people with weakened immunity and KSHV infection, and it is more common in men who have sex with men, likely due to higher KSHV prevalence in this segment of the population. While there is some sexual transmission of the virus, it is typically discovered in saliva rather than the semen. The introduction of highly active antiretroviral therapy (HAART) has significantly altered the landscape of KS and other HIV-related malignancies (1–4).

KS, an AIDS-defining malignancy, typically develops in patients with low CD4 counts (<200 cells/mm^3^) (5–7). Although the incidence of KS has declined over the past decade, it remains uncertain whether KS will now emerge in individuals with higher CD4 counts or if any epidemiologic shifts are underway. Classic KS, commonly affecting older males of Mediterranean, Middle Eastern, and Eastern European descent, is associated with high prevalence of KSHV in the regions. While the exact reasons remain unclear, some studies suggest that communities with high KSHV rates likely contracted the virus as children, probably through saliva transmission from mother to child. Though these individuals may have harbored the virus throughout their lives, the malignancy often develops as a result of a natural, age-related reduction in immune function (8).

The overall burden of KS in equatorial Africa tends to skew toward older populations, as they are more likely to have been exposed to KSHV over a longer period. This demographic trend highlights the need for targeted interventions that consider age-specific vulnerabilities and the ongoing impact of both HIV and KSHV in the region (9). Patients with Kaposi sarcoma, regardless of the form or underlying cause, frequently present with skin lesions that are purple, red, or brown, which can be flat or elevated, diffuse or localized, and often disfiguring – similar to the presentation in this case report. These lesions can also appear in the mouth, anus, or elsewhere in the gastrointestinal tract. If the lesions spread to the lungs, patients may experience symptoms such as cough with a blood-tinged sputum, shortness of breath, and even respiratory failure. Lymph node involvement, particularly in the groin, has been linked to uncomfortable swelling in the legs; as was observed in this case. As with all malignancies, early detection and treatment using available choices can improve symptoms and reduce the likelihood of the disease spreading to other organs. Treatment of KS depends on its subtype, disease extent, and immune status. Localized therapies such as surgical excision, cryotherapy, and radiation are used for early disease, while systemic options include HAART for HIV-positive patients, chemotherapy, and immunomodulatory agents (7, 8). History, appropriate physical examinations with blood tests, and biopsies from the skin, mouth, and lymph nodes aid in the diagnosis of KS (8); these methods were used in this instance.

In our literature review, conducted over three decades using keywords such as “Kaposi sarcoma” and “HIV with high CD4 count,” we identified 14 cases of KS occurring in HIV-positive individuals with high CD4 counts and suppressed viral loads (Supplementary Table 1). This finding underscores the rarity of KS in such clinical settings, despite advancements in HAART, and highlights the significance of documenting these uncommon presentations.

## Case presentation

**Figure 1:**
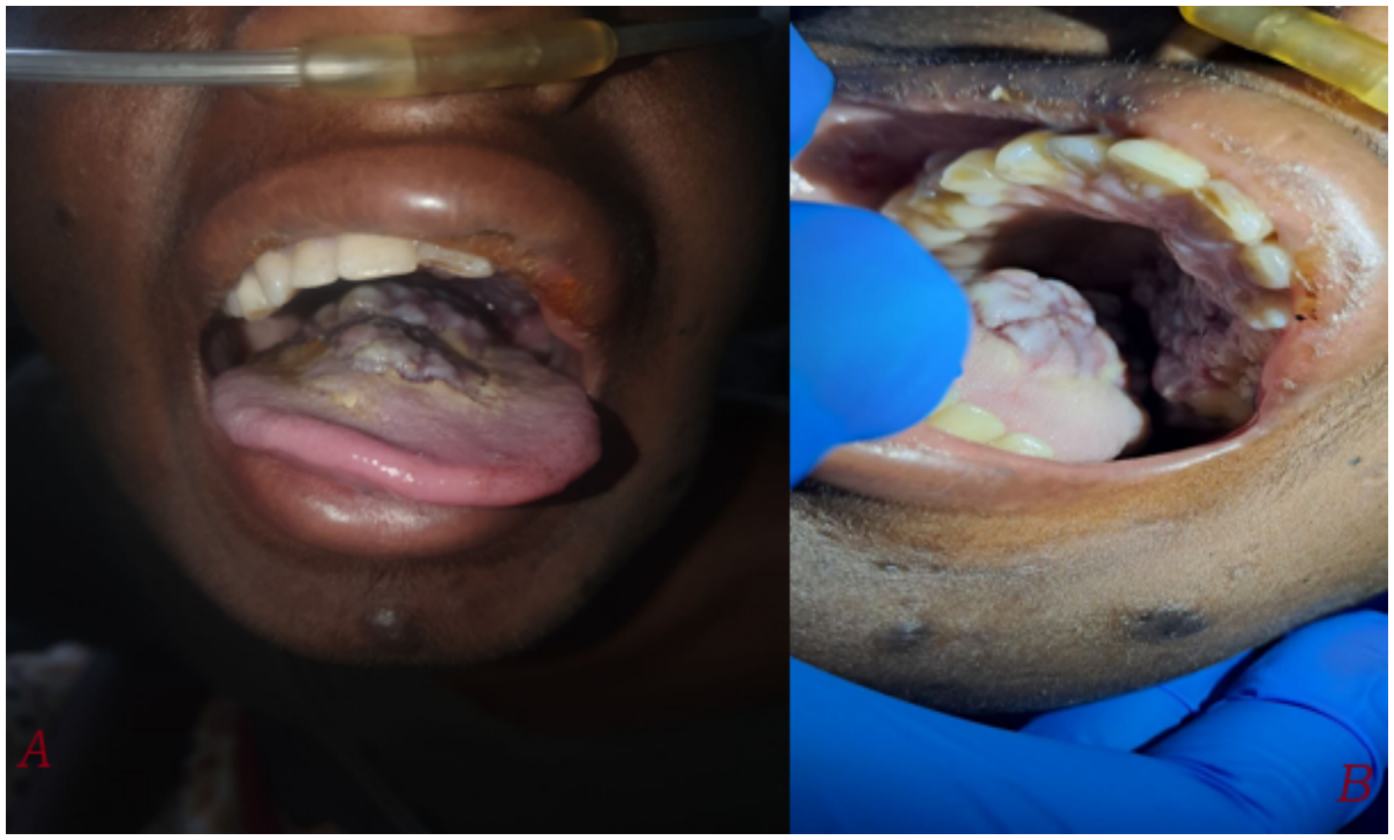
Taken during time of admission on august 21, 2024. **(A)** A large, irregular, bluish-purple exophytic mass measuring approximately 3×3 cm is visible on the posterior tongue, extending to the hard palate. The tongue appears swollen with noticeable discoloration. **(B)** A closer view shows the irregular, lobulated surface of the mass, involving both the hard palate and surrounding soft tissues.

An 18-year-old male from Ethiopia, with a history of vertically acquired HIV infection since birth and currently on dolutegravir/lamivudine/tenofovir (DTG/3TC/TDF) HAART regimen, and a recent CD4 count of 627 cells/mm^3^ and recent viral load of 378 copies/ml, presented on august 21, 2024 with worsening of shortness of breath, cough productive of blood-tinged sputum, fatigue, loss of appetite, and skin lesions of two weeks duration. He also reported tongue swelling, as well as right arm and left leg swelling over the past four weeks. He denied history of fever, chest pain, orthopnea, paroxysmal nocturnal dyspnea, any gastrointestinal symptoms such as diarrhea or abdominal pain. He had no prior history of a similar illness, HIV-related conditions, or any malignancies during his follow-up. Six months ago, he received outpatient treatment for community-acquired pneumonia but had no other infections requiring medical attention. There was no history of allergy, recent sore throat, or recent history of medication changes. He has been consistently taking her HAART medications since he started, with his mother providing strict support to ensure adherence. On examination, the patient was afebrile but exhibited severe respiratory distress. His vital signs revealed blood pressure of 110/70 mmHg, a pulse rate of 116 beats per minute, a respiratory rate of 32 breathes per minute, axillary temperature of 36.8°c, and oxygen saturation of 82% which improved to 93% with supplementation at flow rate of 3 L/min. Conjunctival pallor was also noted. Oral examination revealed a bluish exophytic mass with irregular borders over the posterior tongue extending to the hard palate (Figure 1).

**Figure 2:**
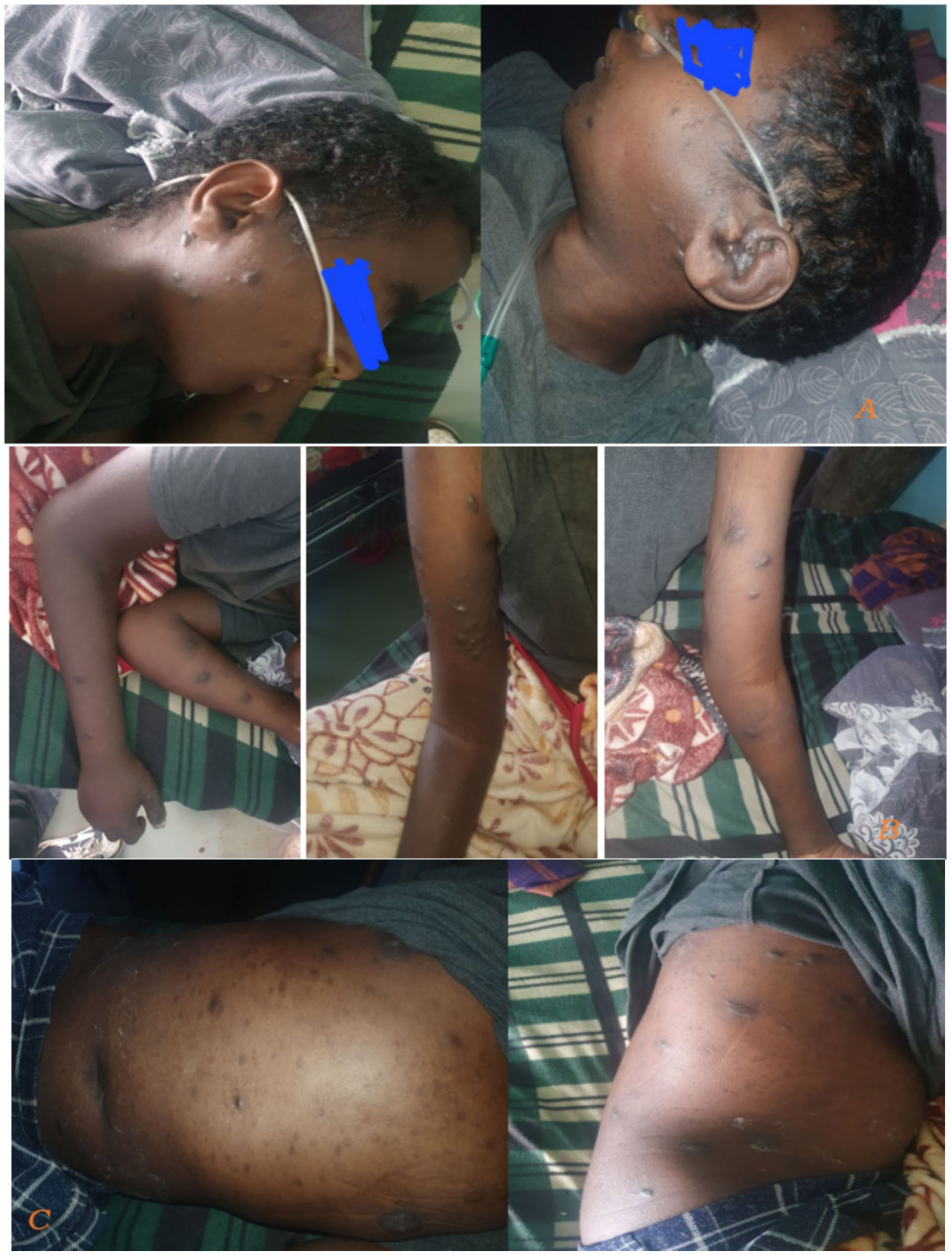
Taken during time of admission on august 21, 2024. There were diffuse hyperpigmented patchy nodular skin lesions involving face, trunk abdomen and extremities.

Skin examination showed diffuse hyperpigmented patchy nodular lesions involving the face, trunk, abdomen and extremities (Figure 2).

There were multiple non-tender, soft lymphadenopathies at the right supraclavicular and submental regions, with the largest measuring 1×1 cm. Respiratory system evaluation revealed bilateral crepitations over the over posterior 2/3rd of the lung fields with some localized wheeze over right intrascapular area. Cardiovascular and abdominal evaluations revealed normal.

**Figure 3:**
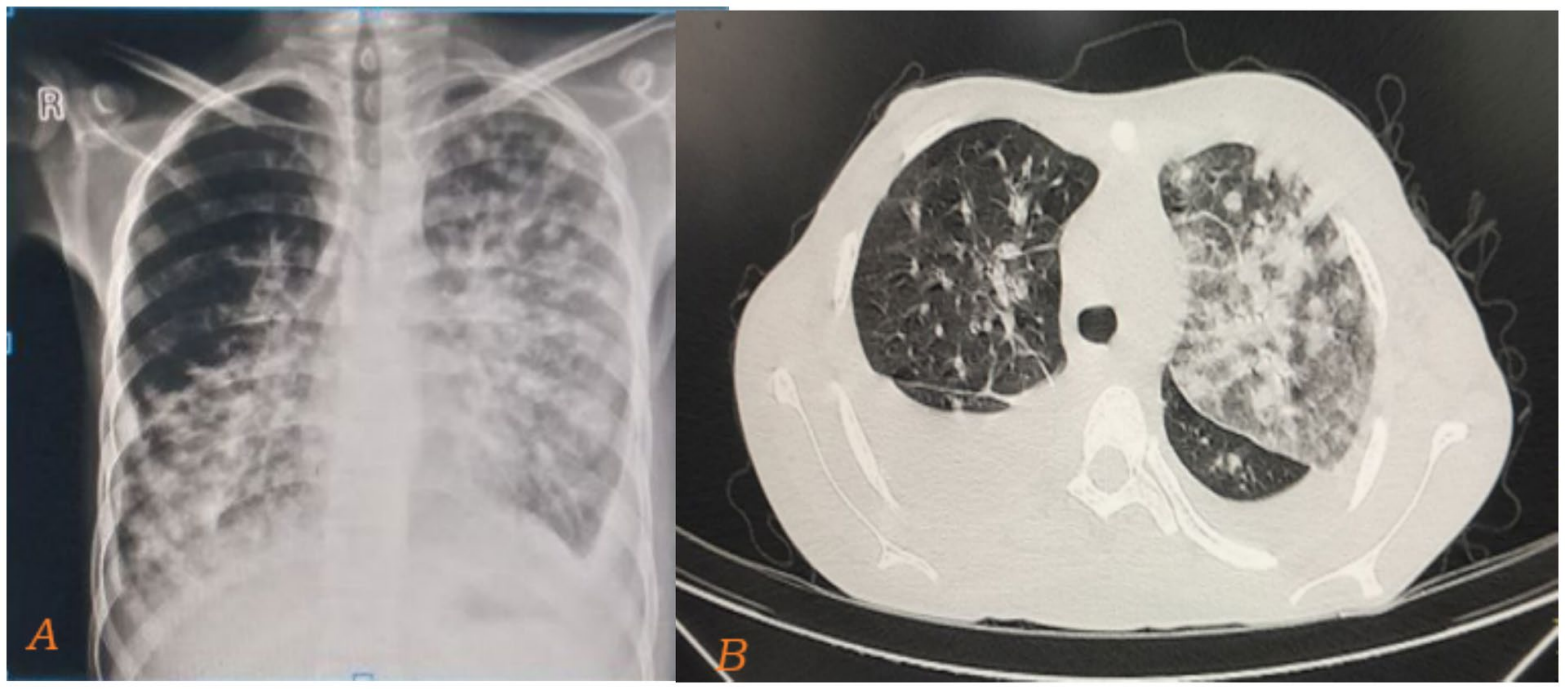
Taken during time of admission on august 21, 2024. Chest X-ray showing bilateral, diffuse, ill-defined, patchy opacities predominantly affecting the mid and lower lung zones **(A)**. The cut slice of the CT scan of the same patient also reveals bilateral, asymmetric, peripherally dominant, ill-defined vascular nodules with a flame-shaped appearance around the bronchi and vessels. These nodules are accompanied by surrounding ground-glass and consolidative opacities, which are suggestive of Kaposi sarcoma **(B)**.

The chest X-ray showed bilateral, diffuse, patchy opacities predominantly affecting the mid and lower lung zones. These opacities are ill-defined and suggestive of an interstitial or alveolar infiltrative process (Figure 3A). The CT scan reveals bilateral, asymmetric, peripherally dominant, ill-defined vascular nodules with a flame-shaped appearance around the bronchi and vessels. These nodules are accompanied by surrounding ground-glass and consolidative opacities, which are suggestive of Kaposi sarcoma (Figure 3B).

**Figure 4:**
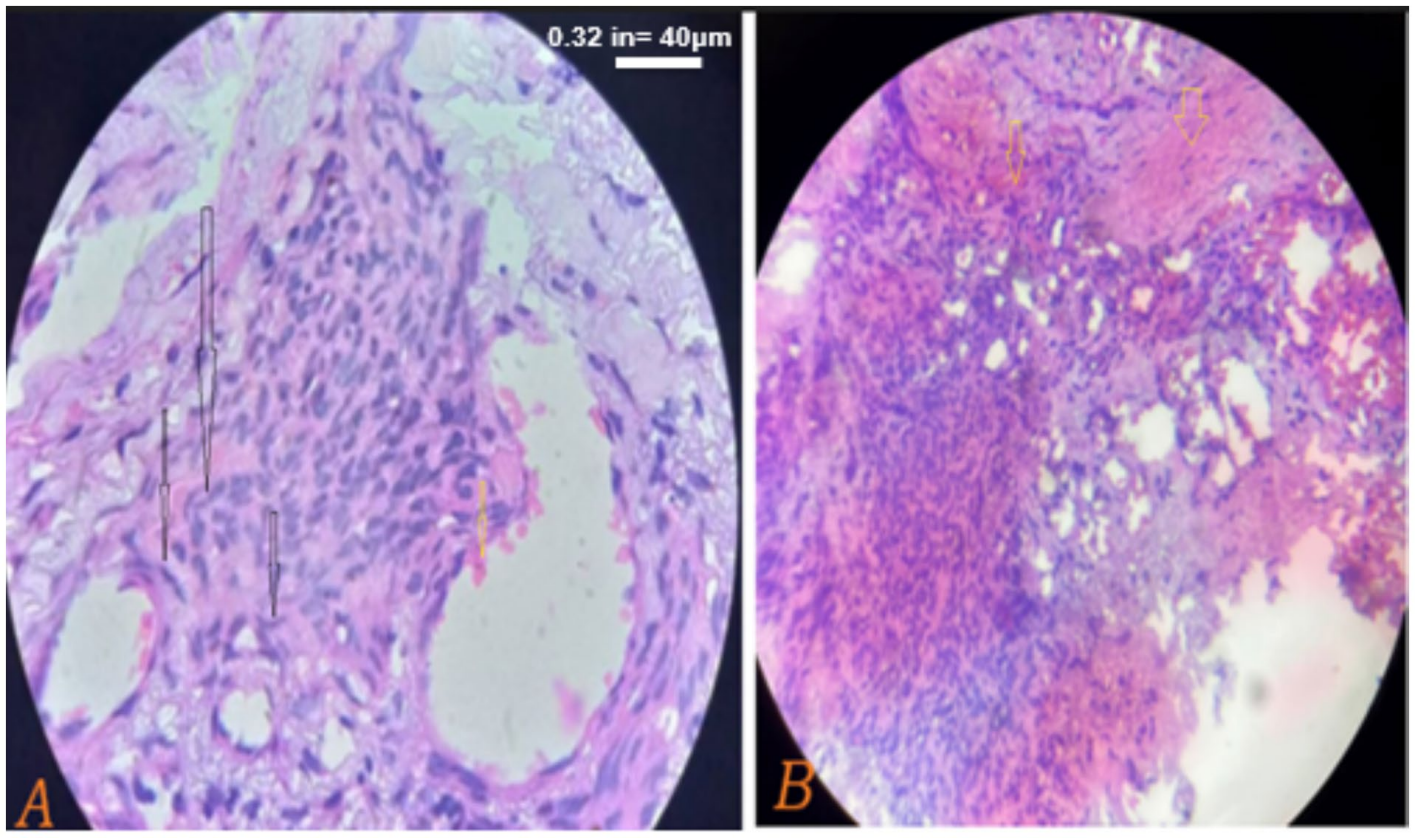
**(A,B)** Taken on his 2nd day post admission on august 22, 2024. Histopathological sections show dermal based sheets of tumoral tissue composed of round spindle cells (black arrows) with hyperchromatic nuclei and inconspicuous nucleoli along with slit-like vascular proliferation and red blood cell extravasations (yellow arrows) beneath an overlying stratified squamous epithelium, consistent with Kaposi sarcoma. The scale bar for both is 40 μm.

Biopsy of the skin, and tongue was highly suggestive of Kaposi sarcoma (Figure 4). Hematological findings included microcytic hypochromic anemia with hemoglobin of 5.8 g/dL, while platelets and white blood cell counts were normal. Serology for viral hepatitis (hepatitis virus B and C) were negative, and liver and renal function tests were within the normal laboratory limits. CD4 cell counts were 627 cell per micrometer, while the viral load was 378 copies per micrometer on the day of presentation. Circulating tumor deoxyribonucleic acid (ctDNA) testing was not performed due to unavailability at the center.

With the diagnosis of HIV-associated Kaposi sarcoma, complicated by severe anemia, the patient was treated with oxygen supplementation, packed red blood cell transfusion, and other supportive care, while continuing the HAART regimen. After some stabilization of the acute symptoms, the patient was advised to continue the multidrug treatment according to the Ethiopian national guideline (10). After 10 days of admission on august 31, 2024 he was also started on paclitaxel 135 mg infusion to be continued every three weeks for a total of six doses, with further follow-up for treatment response advised. On his second week of chemotherapy, the patient developed high-grade fever and cough. With a clinical impression of sepsis of chest origin, he was admitted to the critical care unit, started on broad-spectrum antibiotics, and required respiratory support. Despite all efforts, the patient succumbed to respiratory failure as the disease progressed.

## Discussion

Kaposi sarcoma has historically been associated with low CD4 counts (<200 cells/mm^3^) and AIDS (5, 6). Although the incidence of KS has declined with the widespread use of HAART (7), it remains uncertain if KS can occur at higher CD4 counts. This case demonstrates that KS can present in patients with relatively high CD4 counts, underscoring the need for vigilance in high-risk patients. A study was conducted on the U.S. Military HIV Natural History Study (NHS) (3, 11) to evaluate KS rates and trends in CD4 counts at the time of KS diagnosis among HIV-infected individuals. It revealed that KS rates were significantly higher for time spent with CD4 < 350 cells/μL compared to those with higher CD4 cells.

In our literature review conducted over three decades, the majority of cases involved younger individuals with an average CD4 count of approximately 400 cells/mL. All cases were on HAART with suppressed viral loads and presented with disseminated Kaposi sarcoma (KS), with cutaneous involvement being the most common presentation. Most cases were managed with HAART alone, while two required chemotherapy, one underwent local surgical resection, and another received IVIG. All patients were discharged with clinical improvement (12–15).

In comparison, our case involves the youngest patient, with vertically transmitted HIV, who presented late with disseminated KS affecting both the skin and mucous membranes, potentially involving internal organs. This made the case particularly critical and challenging to manage.

In Equatorial Africa, a notable prevalence of KSHV infection raises questions about the demographic patterns of Kaposi’s Sarcoma (KS), particularly why young boys are predominantly affected while classic KS primarily occurs in older men (16). Patients with KS, regardless of the form or underlying cause, frequently present with skin lesions that are purple, red, or brown, which can be flat or elevated, diffuse or localized, and often disfiguring – similar to the presentation in this case report. These lesions can also appear in the mouth, anus, or elsewhere in the gastrointestinal tract. If the lesions spread to the lungs, patients may experience symptoms such as cough with a blood-streaked sputum, shortness of breath, and even respiratory failure. Lymph node involvement, particularly in the groin, has been linked to uncomfortable swelling in the legs; as was observed in this case. As with all malignancies, early detection and treatment using available choices can improve symptoms and reduce the likelihood of the disease spreading to other organs. History, appropriate physical examinations with blood tests, and biopsies from the skin, mouth, and lymph nodes aid in the diagnosis of KS (8, 17); these methods were used in this instance.

While researches have shown a decline in KS rates during the HAART period, with lower CD4 counts remaining an important risk factor, a significant proportion of KS cases are now emerging at higher CD4 levels. In the late HAART phase, nearly a third of KS cases occurred at CD4 counts ≥350 cells/mm3 (3, 11). Clinicians should be aware of these patterns and remain vigilant for the emergence of KS even with robust CD4 counts. The presented case further underscores that KS can occur at any level of CD4 counts, despite the general trend of improved outcome with HAART.

Although KS has previously been documented at higher-than-expected CD4 levels (10, 18–22), this case is notable as the patient developed KS despite progressive increase in CD4 counts during HAART treatment for HIV. Some studies have linked KS in individuals with high CD4 counts to high rates of drug use and poor adherence to antiretroviral therapy (20, 22). However, in this case, the patient reported excellent adherence to medication and minimal drug use, making it a unique presentation.

Some KS instances in the setting of HAART may be associated to the immune reconstitution inflammatory syndrome (IRIS) (23, 24). However, the reported case has been on HAART for the past 17 years, with no recent regimen changes. Although Kaposi sarcoma (KS) typically presents as less aggressive and more localized with sustained HAART adherence (25), this case demonstrates that KS can still occur in patients with high CD4 counts and present severely, involving multiple organ systems, underscoring the need for continued vigilance despite optimal treatment.

KS remains a significant concern among HIV-infected individuals, despite higher CD4 counts. This case emphasizes importance of healthcare providers maintaining vigilance for the onset of KS, regardless of CD4 levels, to ensure early identification and prompt management, ultimately improving patient outcome.

These patients are managed accordioning to conventional guidelines for AIDS-associated KS, although their treatment needs and tolerance are obviously different. Literature suggests that virally suppressed patients may experience more indolent forms of KS, potentially with visceral involvement, which is rarely fatal (17, 26). To enhance long-term tolerability, treatment strategies should consider lower doses of chemotherapy and extended intervals between treatments. Induction chemotherapy, followed by maintenance or intermittent oral therapy, could be employed to prevent recurrences (17, 27). In this case, the patient was initiated on weight-adjusted Adriamycin along with supportive care, while continuing HAART.

### Strengths and limitations

This case highlights the rare occurrence of Kaposi sarcoma in an HIV-positive patient with a relatively high CD4 count, emphasizing the need for clinical vigilance beyond traditional immunosuppression markers. The detailed clinical, radiological, and histopathological evaluation strengthens the diagnostic accuracy. Despite aggressive management and strict clinical follow-up, the patient succumbed to respiratory failure, underscoring the aggressive nature of advanced Kaposi sarcoma. The lack of circulating tumor DNA (ctDNA) testing and limited access to advanced molecular diagnostics posed challenges in further characterizing the disease. This case reinforces that high clinical suspicion is essential for early diagnosis and intervention to prevent late-stage presentations and mortality.

## Conclusion

Kaposi sarcoma, typically a disease of the immunocompromised, can occur in people living with HIV despite viral loads are low and CD4 counts are near-normal range. Despite advances in HAART, there remains a possibility of advanced KS, necessitating the need for healthcare providers to remain vigilant for its occurrence, even in patients with higher CD4 levels, to ensure timely identification and management. High suspicion and early diagnosis in patients with characteristic lesions are crucial for initiating effective treatment that would improve outcomes.

## Patient perspective

The patient was unable to provide their perspective due to the severity of the illness and subsequent passing. However, their family emphasized the challenges of late diagnosis despite ongoing HIV management and the importance of awareness for similar cases.

## Data Availability

The original contributions presented in the study are included in the article/Supplementary material, further inquiries can be directed to the corresponding author/s.
