# Supplementary material for: Kaposi sarcoma in an HIV-infected patient with high CD4 count: a case report and literature review
Source: Front Med (Lausanne). 2025 Apr 25;12:1496863. doi: 10.3389/fmed.2025.1496863 (PMC12061674; doi:10.3389/fmed.2025.1496863)
Supplement: Supplementary file 1 [file Supplementary_file_1.docx]

| Reference | Year | No. cases | Age/sex | Diagnosis | Serostatus | CD4 count | Treatment | Outcome |
| --- | --- | --- | --- | --- | --- | --- | --- | --- |
| Miller et al. (1999) | 1989 | 1 | 38/M | Cutaneous Kaposi’s sarcoma | + | 400 cell/mL | HAART | improved |
| Miller et al. (1999) | 1999 | 1 | 44/m | Kaposi’s sarcoma | + | 496 cell/mL | HAART + local resection | improved |
| Maurer et al. (2007) ** | 2004-2006 | 9 | range, 41 to 74/NM | Cutaneous Kaposi’s sarcoma | + | >300 cells/mm3 | HAART | improved |
| Lim et al. (2018) | 2017 | 1 | 22/F | disseminated Kaposi’s sarcoma | + | 424/uL | HAART + intravenous liposomal doxorubicin | improved |
| Gupta et al. (2020) | 2020 | 1 | 55/M | disseminated disease | + | 350 cells/mm3 | HAART + paclitaxel | improved |
| Ibrahim et al. (2021) | 2021 | 1 | 20/M | Kaposi’s sarcoma with bone marrow infiltration + ITP | + | 437 cell/mL | HAART + IVIG | improved |
| NM: not mentioned  **: report on a cluster of cases of cutaneous HIV-associated Kaposi's sarcoma occurring in nine patients with high CD4 count. | | | | | | | | |

Supplementary table 1: Presents a comprehensive summary of the literature reviewed from PubMed, using the keywords "Kaposi's sarcoma," "HIV," and "high CD4 count." The review spans an extensive period of over three decades, from 1989 to 2021.
